# Reply to: Challenges in interpreting the role of gentamicin in treatment of invasive listeriosis: immortal timebias and confounding

**DOI:** 10.1007/s15010-024-02417-4

**Published:** 2024-11-25

**Authors:** Jan P. Sutter, Lorenz Kocheise, Jan Kempski, Martin Christner, Dominic Wichmann, Hans Pinnschmidt, Stefan Schmiedel, Ansgar W. Lohse, Samuel Huber, Thomas Theo Brehm

**Affiliations:** 1 I. Department of Medicine, University Medical Center Hamburg-Eppendorf, Martinistraße 52, 20246 Hamburg, Germany; 2 German Center for Infection Research (DZIF), Partner-Site Hamburg-Lübeck-Borstel-Riems, 20246 Hamburg, Germany; 3 Division of Clinical Infectious Diseases, Research Center Borstel, 23845 Borstel, Germany; 4 Institute of Microbiology, Virology and Hygiene, University Medical Center Hamburg-Eppendorf, 20246 Hamburg, Germany; 5 Department of Intensive Care Medicine, University Medical Center Hamburg-Eppendorf, 20246 Hamburg, Germany; 6 Center for Experimental Medicine, Institute for Medical Biometry & Epidemiology, University Medical Center Hamburg-Eppendorf, 20246 Hamburg, Germany

**Dear Editor,**

We would like to thank Hayato Mitaka and Takaaki Kobayashi for sharing their thoughtful comments on our study and their concerns about the conclusions that were drawn in their recent letter “Challenges in Interpreting the Role of Gentamicin in Treatment of Invasive Listeriosis: Immortal TimeBias and Confounding”. Their careful reading gives us the opportunity to clarify several aspects of the manuscript.

The authors raise concerns about the potential for immortal time bias due to the timing of cohort entry, suggesting that patients in the gentamicin group may have survived the most critical phase of infection before receiving the drug. We would like to clarify that our Kaplan-Meier survival analysis was initiated from the time of microbiological diagnosis of invasive listeriosis, rather than from symptom onset or hospital admission. This methodological choice was intended to minimize the risk of immortal time bias. However, we acknowledge that this should have been explained more explicitly in the manuscript to avoid any misunderstanding about the timing of our analysis.

**Table 1:** Results of multivariable Cox regression analyses of the effects of the mortality risk factors gentamicin treatment, neurolisteriosis, chronic kidney disease, sex, and age-adjusted Charlson Comorbidity Index (aaCCI) on 90-day mortality. Hazard ratio (HR), confidence interval (CI)

Analysis		HR	95%CI for HR	*p*-value
1	Gentamicin combination treatment	0.05	0.01–0.45	0.01
	aaCCI	0.16	0.02–1.43	0.10
	Chronic kidney disease	1.31	1.03–1.68	0.03
	Male sex	1.71	0.34–8.64	0.52
	Neurolisteriosis	2.32	0.34–15.90	0.39
2	Gentamicin combination treatment	0.07	0.01–0.51	0.01
	Propensity score	3.13	0.21–46.03	0.41

Secondly, the authors raised the issue of renal function and its potential role as a confounding factor. They correctly noted that there were numerically more patients with chronic kidney disease in the monotherapy group than in the gentamicin group. However, we specifically analyzed creatinine levels at the time of diagnosis in both groups and found no significant differences. We deliberately chose not to exclude patients with elevated renal function parameters or kidney disease from our analysis, as these conditions are not absolute contraindications to gentamicin treatment. While both acute and chronic kidney injury require careful consideration, gentamicin can still be used, with appropriate dose adjustments and close monitoring, as part of a personalized treatment approach on a case-by-case basis.

The Pearson Chi-Square test showed no significant association between gentamicin treatment and the development of chronic kidney disease (CKD) (data not shown). When chronic kidney disease was included in the multivariable Cox regression analysis, its effect was weakly significant. However, the treatment effect of gentamicin changed only marginally, with the hazard ratio slightly decreasing, from 0.6 in the previous analysis to 0.5 in the CKD-adjusted analysis. This indicates that the combination of gentamicin treatment had an even stronger positive effect on outcomes when chronic kidney disease was accounted for in the model (Table 1, analysis 1). Using a propensity score (based on all covariates other than gentamicin treatment, including CKD) instead of individual covariates in the model did likewise change the treatment effect only marginally, as compared to the previous analysis (Table 1, analysis 2). Therefore, we believe that the conclusions regarding the beneficial effect of gentamicin combination therapy in our cohort remain valid.

We agree with the authors that future studies could be strengthened by incorporating additional methodological approaches, such as matching based on the timing of treatment initiation. Although prospective studies would avoid these limitations, they are difficult to conduct in diseases with relatively small case numbers, like invasive listeriosis.

Despite the limitations of our study, we are confident that it offers valuable insights into the potential benefits of gentamicin combination treatment for invasive listeriosis, and we hope our clarifications sufficiently address the concerns raised.

Thank you for the opportunity to engage in this important discussion.

## Data Availability

No datasets were generated or analysed during the current study.

